# Leg orientation as a clinical sign for pusher syndrome

**DOI:** 10.1186/1471-2377-6-30

**Published:** 2006-08-23

**Authors:** Leif Johannsen, Doris Broetz, Hans-Otto Karnath

**Affiliations:** 1 Section Neuropsychology, Center of Neurology, Hertie-Institute for Clinical Brain Research, University of Tübingen, Tübingen, Germany; 2 Behavioural Brain Sciences Centre, School of Psychology, University of Birmingham, Birmingham, UK

## Abstract

**Background:**

Effective control of (upright) body posture requires a proper representation of body orientation. Stroke patients with pusher syndrome were shown to suffer from severely disturbed perception of own body orientation. They experience their body as oriented 'upright' when actually tilted by nearly 20° to the ipsilesional side. Thus, it can be expected that postural control mechanisms are impaired accordingly in these patients. Our aim was to investigate pusher patients' spontaneous postural responses of the non-paretic leg and of the head during passive body tilt.

**Methods:**

A sideways tilting motion was applied to the trunk of the subject in the roll plane. Stroke patients with pusher syndrome were compared to stroke patients not showing pushing behaviour, patients with acute unilateral vestibular loss, and non brain damaged subjects.

**Results:**

Compared to all groups without pushing behaviour, the non-paretic leg of the pusher patients showed a constant ipsiversive tilt across the whole tilt range for an amount which was observed in the non-pusher subjects when they were tilted for about 15° into the ipsiversive direction.

**Conclusion:**

The observation that patients with acute unilateral vestibular loss showed no alterations of leg posture indicates that disturbed vestibular afferences alone are not responsible for the disordered leg responses seen in pusher patients. Our results may suggest that in pusher patients a representation of body orientation is disturbed that drives both conscious perception of body orientation and spontaneous postural adjustment of the non-paretic leg in the roll plane. The investigation of the pusher patients' leg-to-trunk orientation thus could serve as an additional bedside tool to detect pusher syndrome in acute stroke patients.

## Background

Posture and balance impairment is frequent in stroke patients. The deficit might be caused either by sensory loss, the presence of contralateral hemiparesis or by affected internal processes of sensory integration and postural control. Hemiparetic stroke patients may show impaired postural stability of the trunk [1], abnormal anticipatory muscle activity in response to oscillations of the support base [2], impaired postural adjustments during voluntary limb movements [3,4], increased sway and prolonged stabilization following external disturbances to the hip [5], disordered temporal sequences of muscle activity when going onto tiptoes [6], delayed muscle response onset latencies following external perturbations [7-9] and asymmetric stance and gait [10-13].

While hemiparesis is the most prominent cause of postural instability in stroke patients, some patients may show a specific postural disorder, which has been termed "pusher syndrome". Different from patients who exclusively suffer from hemiparesis, these patients use the non-hemiparetic arm and/or leg to push away actively from the non-paralyzed side [14,15]. Without assistance, this active pushing leads to loss of postural balance and falling towards the paralyzed side. Nevertheless, the patients resist any attempt to correct passively their tilted body posture towards the earth-vertical upright orientation. It turned out that patients with pusher syndrome suffer from a severely altered perception of the body's orientation in relation to gravity. They experience their body as oriented 'upright' (subjective postural vertical; SPV) when actually tilted near 20° to the ipsilesional side [16]. It turned out that pusher syndrome characteristically is provoked by unilateral left or right brain lesions in the posterior thalamus [17,18] and less frequently in the insula and postcentral gyrus [19].

Keeping a stable body posture against both gravitational force and other perturbations of balance requires a postural control system to regulate the muscles of all body segments in a coordinated fashion. Also, a representation of own body position with respect to the earth-vertical is needed to drive the appropriate postural adjustments in response to perturbations of balance [20,21]. Observation of the spontaneous posture of the body segments in the seated subject may be a reasonable approach to predict the subject's subjective postural vertical (SPV) as it is computed from somatosensory afferent information. Evidence has been reported that during a seated posture, when the base of contact is wide, somatosensory afferent information is used to generate a body-centered representation of verticality that drives the appropriate postural responses. Tilt of the body causes postural responses of all body segments and especially the legs [22,23]. The sensory sources used to guide these stabilizing movements depend on the specific environmental context and does not seem to be a function of exclusively the vestibular system. Somatosensory afferences sometimes appear more important for postural control than visual or vestibular input [24-28].

The examination of pusher patients' SPV by using a tilt chair apparatus [16] is burdened with some disadvantages for daily clinical practice on the ward. For example, the patients are required to keep focussed attention over a relatively long investigation period in order to give consistent responses. This can be problematic in stroke patients, especially in the acute phase after stroke onset. The aim of our study thus was to investigate pusher patients' subjectively perceived body orientation directly at the bedside. We asked whether it is possible to derive this measure quasi "indirectly", i.e. without the patients' explicit collaboration and attention, simply through their spontaneous postural adjustments. We focused on the spontaneous head and leg posture in seated patients with pusher syndrome during passive body tilt in the roll plane using kinematic methods. In order to investigate the impact of vestibular input to postural adjustments of the head and the leg in seated subjects, we contrasted the pusher patients' behaviour with a group of patients suffering from acute unilateral vestibular loss due to neuritis or surgery.

## Methods

We investigated 9 acute stroke patients with severe pusher syndrome and 7 patients showing an acute unilateral vestibular loss. In addition, 9 acute stroke patients without pushing behaviour or vestibular dysfunction, as well as 10 non-brain-damaged control subjects were examined (cf. Table 1). Stroke patients were included if they demonstrated an acute unilateral brain lesion by magnetic resonance imaging (MRI) or by computed tomography (Spiral-CT). Patients with diffuse or bilateral brain lesions or patients with tumors were excluded.

We matched our four groups of subjects as closely as possible concerning demographic and clinical characteristics. Still, with respect to the age of the subjects almost significant differences were present in our sample (F(3,31) = 2.86, p = 0.053). Post-hoc Scheffé comparisons revealed that this was due to a tendency of the group of pusher patients to be older than the vestibular patients (p = 0.062). The time since onset of the disorder and the present investigation tended to be different between the three groups of patients with acute disorders but also failed significance (F(2,22) = 2.65, p = 0.093). Comparisons between the two groups of stroke patients with or without pusher syndrome revealed that the frequency of spatial neglect was higher in case of the pusher patients (χ^2 ^= 5.33, p < 0.05), while the frequency of aphasia was greater in the patients not showing contraversive pushing (χ^2 ^= 4.57, p < 0.05). The frequency of visual field defects (χ^2 ^= 0.00, p > 0.05) as well as the frequency of contralateral somatosensory loss (χ^2 ^= 0.00, p > 0.05) were comparable between the pusher patients and the control stroke patients without pushing behaviour. Paresis was more severe in the stroke patients with pusher syndrome than in those without, for the arm and the leg (both U ≥ 4.5, both p ≤ 0.027).

All subjects or their relatives gave their informed consent to participate in the study, which was performed in accordance with the ethical standards laid down in the 1964 Declaration of Helsinki and approved by the local research ethics committee. Demographic and clinical data of all subjects are presented in Table 1.

### Clinical investigation

In all three patient groups, pusher syndrome was assessed by a trained physiotherapist (D.B.) using the standardized 'Scale for Contraversive Pushing' (SCP) [15,16]. The SCP assesses (i) symmetry of spontaneous posture while sitting and standing, (ii) the use of the non-paretic arm and/or leg to increase pushing force by abduction and extension of extremities while sitting and standing, and (iii) resistance to passive correction of posture while sitting and standing. Pusher syndrome was diagnosed if all three criteria were present and when the patients showed at least a total score of 1 (max. = 2, sitting plus standing) with respect to their spontaneous posture, at least a score of 1 (max. = 2, sitting plus standing) concerning the use of the non-paretic arm and/or leg to increase pushing force by abduction and extension, and when they showed resistance to passive correction of posture. (In four stroke patients, pushing behaviour while standing could not be quantified with SCP due to a complete inability to reach a standing position at the time of the investigation.) Further details of the SCP assessment are presented in Table 1.

Visual field defects were assessed by standard neurological examination. The degree of paresis of the upper and lower limbs was scored with the usual clinical ordinal scale, where '0' stands for no trace of movement and '5' for normal movement. Spatial neglect was diagnosed when the patients showed the characteristic clinical behavior such as orienting towards the ipsilesional side when addressed from the front or the left and/or ignoring of contralesionally located people or objects. In addition, all patients were further assessed with the "Letter cancellation" task [29], the "Bells test" [30], the "Baking tray task" [31], and a copying task [32]. Neglect patients had to fulfill the criterion for spatial neglect in at least two of these four clinical tests [33]. Aphasia was assessed conducting a bedside examination that evaluated spontaneous speech, auditory and reading comprehension, picture naming, reading, and oral repetition.

The patients with acute unilateral vestibular loss (neuritis, surgery) were admitted with acute vertigo, vomiting and dizziness. They showed severe problems of keeping balance during walking and upright stance with open and closed eyes. The diagnosis of acute unilateral vestibular loss was further based on the following electronystagmographic findings: horizontal spontaneous nystagmus, ability to suppress the spontaneous nystagmus by fixation, no unilateral vestibulo-ocular response to stimulation by rotation of the chair (120°/s) and to unilateral caloric stimulation with warm (44°C), cold (30°C) and ice (3°C) water. Additionally, the subjective visual vertical (SVV) was determined. The selected patients showed an average tilt of the subjective visual vertical (SVV) of 5.0° (SD 3.9°) to the side of the vestibular loss. MR-imaging was performed to exclude ischemia, tumor or any other lesion of the central nervous system in the patients with vestibular loss.

### Experimental protocol and apparatus

Subjects sat at the bedside with legs hanging free and feet off the ground. They were stabilized by one experimenter standing on the patients' contralesional side or in case of the non brain damaged controls on the left side. The subjects were instructed that they would be moved sideways and that they should not resist this passive movement. They were further instructed to keep arms on their lap and to look into the direction of the camera such that they could easily be recorded from the front (see below). The experimenter applied a smooth and slow sideways tilting motion of the subjects' trunk to the left and right. Subjects were tilted under two viewing conditions for five cycles each, first with open eyes and then while being blind-folded.

White, circular markers (diameter 3 cm) were attached to the subject's body. One marker was placed on the subjects' forehead center, one at the chin, one at the solar plexus, and one at the navel. The first two markers indicated the orientation of the head, the latter two the orientation of the trunk. Two further markers were used to determine the orientation of the lower leg. In stroke patients the markers were attached to the ipsilateral, non-paretic lowerleg. In the non brain damaged control subjects, they were attached to the right lower leg; in the case of acute vestibular patients to the side ipsilateral of the vestibular loss. Marker positions were recorded with a digital camcorder (Sony DCR-HC14E) in a normal lighted room with a sample rate of 30 Hz. The camera was placed at a distance of 3 meters right in front of the subject at a height of 1.20 meters which corresponded to the subject's upper chest. The whole data collection procedure took no longer than ten minutes and usually was performed at the beginning of a regular physiotherapy treatment session.

### Data analysis

The white body markers were tracked frame-by-frame using Winanalyze V1.4 (Mikromak), which resembles an automatized software for tracking visual markers in video files. Marker positions were reset manually by one experimenter (L.J.) whenever the software lost its' track. Final marker positions were analyzed using Matlab 6.5 (The Mathworks). Tilt angles of the head, trunk and leg were determined for each single frame. Positive values indicated a rightward or an ipsiversive tilt of the head, trunk or leg with respect to the earth-vertical; negative values a leftward or contraversive tilt. Subsequently, we determined the relative angles of the two segments by calculating the orientation of both the head and the leg with respect to the trunk orientation. Data were averaged across the tilt range for each tilt direction respectively. For each variable, we performed three-way repeated-measures analyses of variance with patient group as the between subject factor and viewing condition and tilt direction as within subject factors. To further analyze significant interactions, we computed additional one- or two-way analyses of variance. For each ANOVA, post-hoc Scheffé comparisons with an α-level of 0.05 were computed between the groups.

To characterize the passive trunk tilt, we determined the maximum trunk tilt range for each individual and performed a fast fourier transformation (FFT) to reveal the frequency of the spectral peak of trunk tilt across the five tilt cycles for each of the two viewing conditions. To obtain trunk tilt velocity (°/sec), we differentiated trunk orientation across two successive frames. The average velocity curve across the five tilt cycles was smoothed with a moving average filter spanning five frames (167 ms). The velocity curve was segmented into individual cycles of movement direction by each zero-crossing. For each direction segment and under both viewing conditions, we determined the maximum trunk velocity.

## Results

### Characteristics of passive trunk tilt

Since the passive trunk tilt in the roll plane was applied manually by an experimenter, we analyzed the characteristics of the body tilt across patient groups. No effects of viewing condition were found for the tilt range, tilt frequency, or maximum tilt velocity (cf. Table 2).

Due to their strong resistance against passive body tilt when crossing the midline into the ipsiversive direction, the pusher patients demonstrated a smaller average tilt range into the ipsiversive direction compared to the other three control groups (post-hoc comparisons: all p ≤ 0.07). No differences were found between the viewing conditions or between the three subject groups without pusher syndrome. Concerning average tilt frequency, no differences were evident between the four subject groups or the viewing condition. As the interaction between group and viewing condition was significant, we performed separate post-hoc comparisons for both the open and the closed eyes conditions. We found no significant differences (all p ≥ 0.14). Finally, maximum tilt velocity was statistically comparable between the four groups of subjects (all p ≥ 0.40). The results indicate that except for the smaller average tilt range for the patients with pushing behaviour, the characteristics of the passive tilt movement was comparable across all four subject groups.

### Spontaneous leg-to-trunk posture

As the pusher patients were tilted in a smaller range into the ipsiversive direction compared to the other three control groups, we restricted the analysis of the leg-to-trunk orientation (same for the head-to-trunk orientation) to the maximum body tilt angles which were reached by each single participant under both viewing conditions. Thus, the body tilt angles which were analyzed for all subjects ranged from -18.3° (contraversive) to 4.3° (ipsiversive) of body tilt.

**Figure 1:**
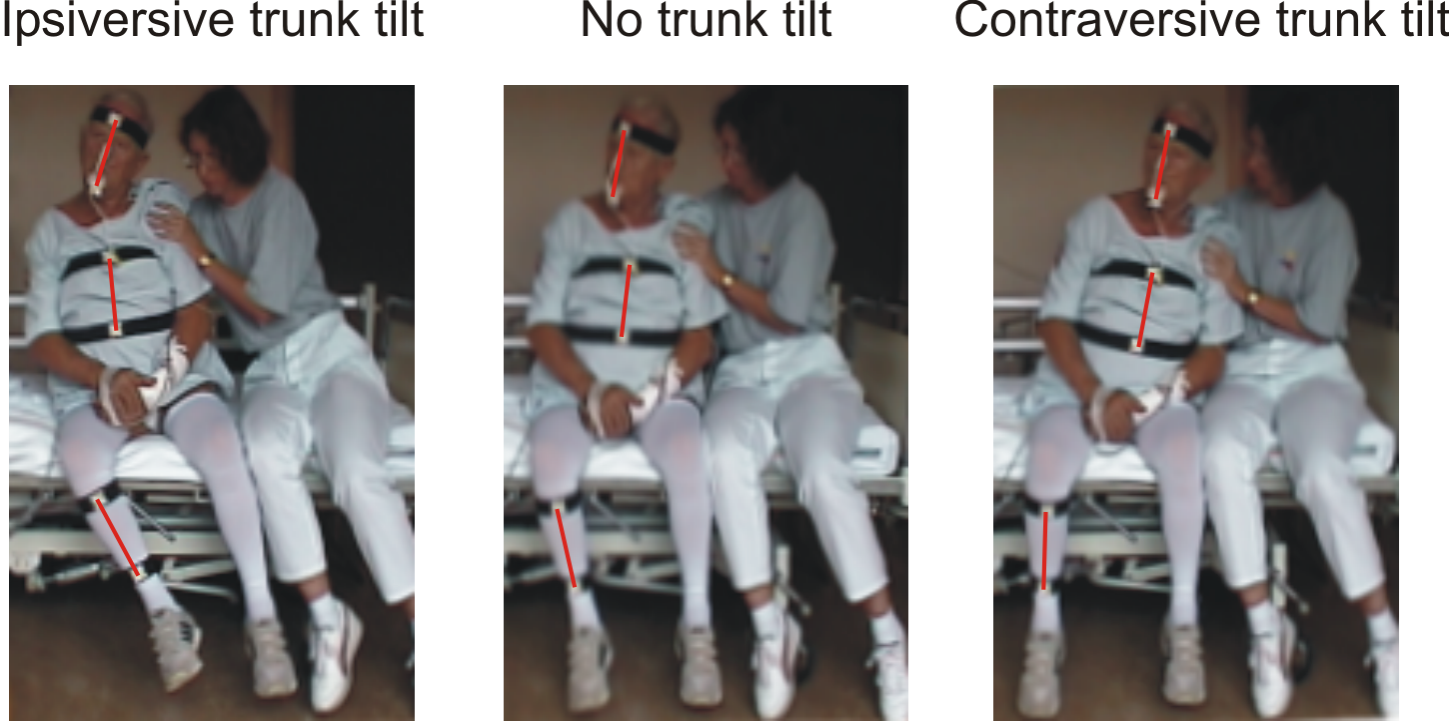
Pusher patient with right hemisphere stroke and left-sided hemiparesis during three tilt positions within one experimental body tilt cycle (open eyes condition). One can easily see that the patient with pusher syndrome exhibits a constant ipsiversive tilt (with respect to trunk orientation) of his non-paretic leg when sitting upright. This inclined leg position was kept throughout the whole tilt cycle. The read lines represent the longitudinal axes of the respective body segments. (The patient does not show pushing behaviour on this series of photos because he was required to hold his arms in his lap and was not able to touch the floor with his feet.)

**Figure 2:**
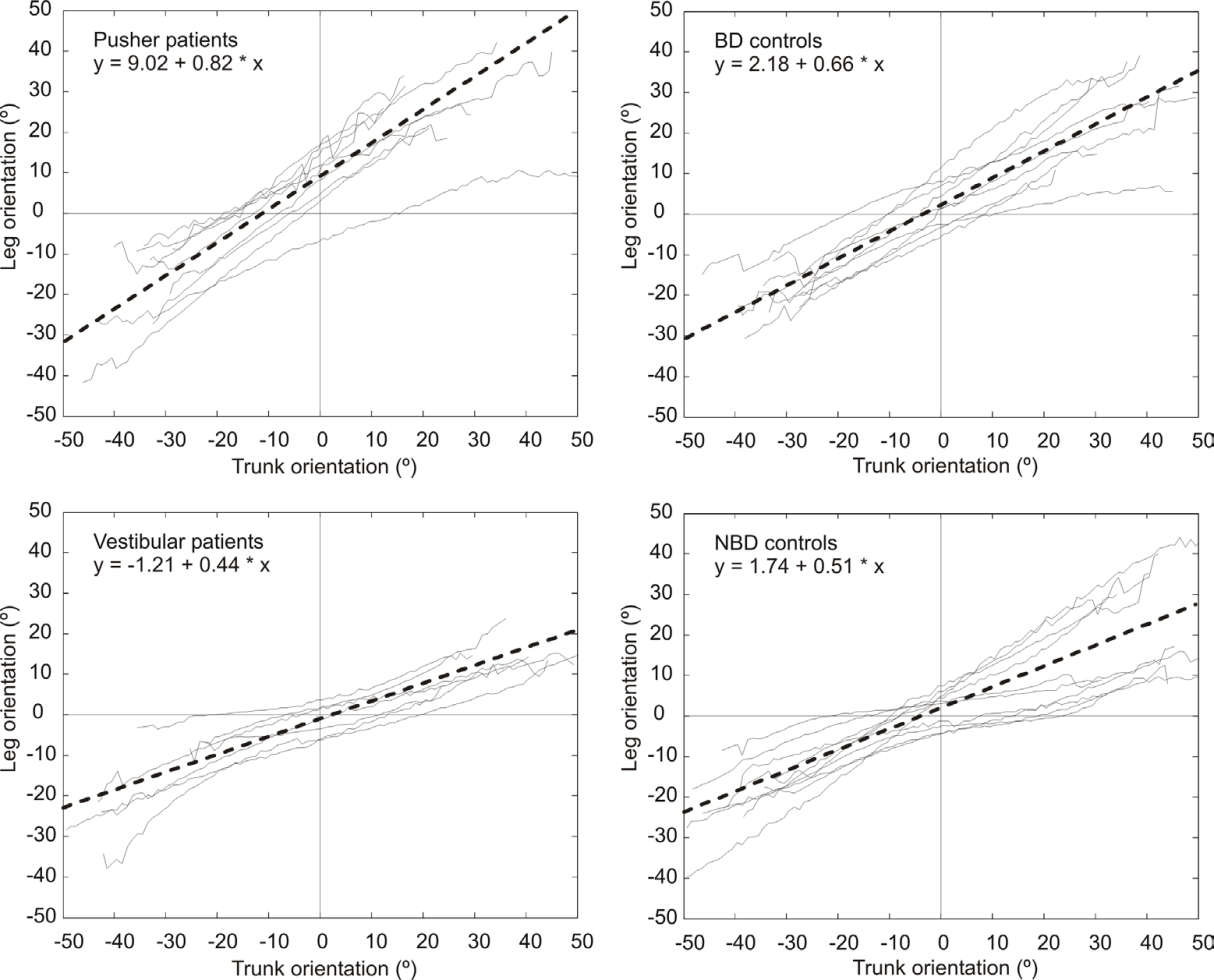
Mean leg orientation as a function of trunk orientation for each subject of all four groups over the whole tilt cycle. Each thin line represents the performance of a single individual, averaged across five tilt cycles and both viewing conditions. The broken thick line indicates each group's averaged linear regression derived from all individuals' regression parameters. Positive values indicate orientation into the ipsiversive/right direction; negative values indicate orientation into the contraversive/left direction.

Figure 1 gives an example of the behaviour of a pusher patient. He shows a constant ipsiversive tilt of his non-paretic leg (with respect to trunk orientation) when sitting upright as well as during the whole tilt cycle. Figure 2 illustrates the orientation of the leg as a function of trunk tilt for each individual subject of all four groups over the whole tilt cycle. We found significant differences for the average leg-to-trunk orientation over the range of body tilt between the four groups of subjects (F(3,31) = 4.65, p = 0.008). Post-hoc comparisons revealed significant differences between the pusher patients' and the vestibular patients' average leg-to-trunk orientation (p = 0.018), while the difference between the pusher patients and the two other groups did not reach significance (BD-controls: p = 0.078; NBD-controls: p = 0.083). In general, the pusher patients demonstrated a pathological, ipsilateral deviation of their average leg-to-trunk orientation of about 6.9°. Viewing condition did not influence the average leg-to-trunk orientation (F(1,31) = 0.15, p = 0.70). Also, the interaction between group and viewing condition was not significant (F(1,31) = 0.84, p = 0.48). Tilt direction significantly influenced average leg-to-trunk orientation (F(1,31) = 117.96, p < 0.001) and significantly interacted with the group factor (F(3,31) = 11.30, p < 0.001). The interaction between viewing condition and tilt direction was also significant (F(1,31) = 4.62, p = 0.039) as well as the interaction between all three factors (F(3,31) = 4.62, p = 0.009).

**Figure 3:**
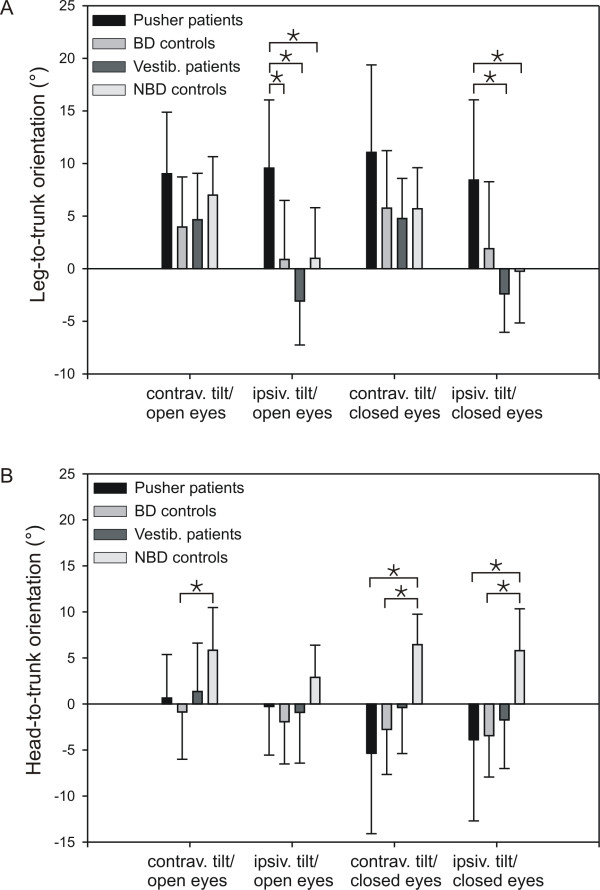
(A) Mean leg-to-trunk orientation of all four subject groups for both directions of body tilt and both viewing conditions. Positive values indicate a relative orientation of the leg into the ipsiversive/right direction; negative values into the contraversive/left direction. (B) Mean head-to-trunk orientation of all four subject groups for both directions of body tilt and both viewing conditions. Error bars indicate standard deviations; asterisks indicate significant differences with p < 0.05.

Figure 3a presents the average leg-to-trunk orientation for all four groups during passive body tilt into both directions under both viewing conditions. Post-hoc comparisons revealed significant differences between the pusher patients and the other three patient groups during body tilt in the ipsiversive direction with open eyes (all p ≤ 0.018). With closed eyes during body tilt in the ipsiversive direction, the pusher patients' average leg-to-trunk orientation significantly different just from the vestibular patients and the NBD-controls (both p ≤ 0.031). No further comparisons were significant. Two additional paired samples t-tests confirmed that the pusher patients' as well as the BD-controls average leg-to-trunk orientation during ipsiversive body tilt was not affected by viewing condition (both t ≤ 0.82, both p ≥ 0.44). This indicates that the patients with pushing behaviour showed an abnormal average ipsiversive leg-to-trunk orientation of about 9.0° (SD 6.5) when passively tilted into the ipsiversive direction irrespective of visual feedback during the body tilt.

From Figure 2 it is evident, that the pusher patients demonstrated an ipsiversive shift of the intercept as well as a steeper slope of the linear regression of leg orientation as a function of trunk tilt. As for the small tilt ranges (especially into the ipsiversive/rightward direction) and to further describe the leg orientation depending on body orientation, we computed the linear regression parameters for the absolute orientation of the leg as a function of the absolute trunk orientation for each individual and for each body tilt direction and viewing condition. A fit of the linear regression was accepted, if R^2 ^exceeded the value of 0.75. Median R^2 ^across all individuals and all conditions was high (0.97; range 0.79 to 0.99). For two pusher patients the linear regression parameters could be estimated only for the contraversive side, as goodness of linear fit was below our criterion of 0.75 during body tilt into the ipsiversive direction. Also, one vestibular patient and one NBD-control subject had to be excluded from further analysis because they did not show a sufficient linear fit during body tilt into a particular direction. Table 3 shows the effect table for the analysis of the linear regression parameters for the leg-to-trunk function.

We found a significant effect of the factor group regarding both the intercept and the slope of the linear regression of the leg-to-trunk function. No further main factor and no interaction became significant. Post-hoc comparisons for the intercept parameter revealed significant differences between the pusher patients and all three groups of subjects without pushing behaviour (all p ≤ 0.029). Post-hoc comparisons for the slope parameter showed significant differences between the pusher patients and both the vestibular patients and the NBD-controls (both p ≤ 0.002) and a tendency for the pusher patients to differ from the BD-controls (p = 0.104) indicating the the pusher patients tended to show an increased slope of the leg-to-trunk function. No further comparisons were significant. Thus, we averaged each of the linear regression parameters across the brain damaged controls, the vestibular patients and the non brain damaged controls and across both viewing conditions. From this we determined the relationship between trunk orientation of the pusher patients and the controls for a given leg orientation. For example, while sitting upright the pusher patients demonstrated a spontaneous orientation of the ipsilateral leg that was shown by the non-pusher subjects when tilted on average about 15° to the ipsiversive side.

### Spontaneous head-to-trunk posture

Analysis of the spontaneous head-to-trunk posture showed a significant effect of group (F(3,31) = 5.79, p = 0.003), a tendency for an effect of viewing condition (F(1,31) = 3.88, p = 0.058), a significant interaction between group and viewing condition (F(3,31) = 3.40, p = 0.03), a tendency for an effect of tilt direction (F(1,31) = 3.36, p = 0.08), no interaction between group and tilt direction (F(3,31) = 0.75, p = 0.53), a significant interaction between viewing condition and tilt direction (F(1,31) = 11.71, p = 0.002), and no interaction between group, viewing condition and tilt direction (F(3,31) = 1.36, p = 0.28). Figure 3b presents the average head-to-trunk orientation for all four groups during passive body tilt into both directions under both viewing conditions. Post-hoc comparisons revealed a significant difference between the BD-controls and the NBD-controls during body tilt in the contraversive/leftward direction with open eyes (p= 0.049). With closed eyes during body tilt into both directions, the NBD-controls' average head-to-trunk orientation was significantly different for the pusher patients' and the BD-controls' average head-to-trunk orientation (all p ≤ 0.023). Additional paired samples t-tests were performed to investigate whether the pusher patients were influenced by the viewing condition. Only during body tilt into the contraversive direction did the pusher patients adopt an increased contraversive tilt of their head-to-trunk orientation with closed eyes (t(8) = 2.43, p = 0.041). The comparison for body tilt into the ipsiversive direction was not significant (t(8) = 1.47, p = 0.18). The opposite was the case for the NBD-controls, they showed an rightward increase of head-to-trunk orientation during rightward body tilt with closed eyes (t(8) = -2.34, p = 0.044) but not during body tilt into the leftward direction (t(8) = -0.56, p = 0.59).

We computed the linear regression parameters for the absolute orientation of the head as a function of the absolute trunk orientation for each individual and for each body tilt direction and viewing condition. Data from three pusher patients were excluded from this analysis because goodness of linear fit was below our criterion of 0.75 during body tilt into the ipsiversive direction. Table 4 shows the effect table for the analysis of the linear regression parameters for the head-to-trunk function.

We found a significant effect of the factor group regarding the intercept of the linear regression of the head-to-trunk function. No further main factor and no interaction became significant with respect to the intercept. Post-hoc comparisons for the intercept parameter revealed significant differences between the BD-controls and the NBD-controls (p = 0.014). For the slope parameter of the head-to-trunk function, we found no effect of group but of viewing condition. The average slope parameter of the head-to-trunk function over all four groups with open eyes was 0.90 (SD 0.27), while it was 0.96 (SD 0.25) with closed eyes. In order to break up the significant interaction between group factor and body tilt direction, we computed two-way repeated-measures analyses of variance on viewing condition and tilt direction for each subject group separately. The pusher patients showed a significant effect of tilt direction (F(1,5) = 11.06, p = 0.02) because of an increased slope (greater than unity) during body tilt into the ipsiversive direction (cf. Table 4) meaning that the tilt of the head was even larger than the trunk tilt. The vestibular patients tended to show the opposite pattern with a greater slope of the head-to-trunk function during body tilt into the contraversive direction (F(1,5) = 3.85, p = 0.097). Interestingly, the vestibular patients were the only group that showed an effect of viewing condition (F(1,6) = 12.72, p = 0.012) which indicated that they kept their head slightly more stable on the trunk with eyes closed (mean = 0.75, SD 0.17) compared to the eyes open (mean = 0.88, SD 0.16).

## Discussion

We investigated the spontaneous postural responses of the head and the non-paretic leg during slow passive body tilt in the roll plane in stroke patients with and without pusher syndrome, in patients with acute unilateral vestibular loss, and in non brain damaged subjects. Especially during body tilt into the ipsiversive direction, the pusher patients showed a markedly different spontaneous leg-to-trunk orientation compared to all three groups without pushing behaviour. Their leg-to-trunk relation was distorted by a constant ipsiversive tilt of the non-paretic leg (with respect to trunk orientation). For example, when seated upright without contact to the ground, an ipsiversive tilt of the non-paretic leg of about 9° was observed. In subjects not showing pushing behaviour, such an orientation of the leg was observed when they were tilted 15° to the ipsiversive side. Moreover, we found the balance response of the pusher patients' non-paretic leg more pronounced compared to subjects without pushing behaviour.

Concerning head orientation during passive trunk tilt, the results were less clear. With open eyes, it seemed that the NBD-controls behaved different from the other three subject groups by adopting an average rightward tilt of the head with respect to the trunk. With closed eyes during rightward passive body tilt, the NBD-controls demonstrated an increase of the rightward tilt of head posture opposite to the pusher patients, who showed a contraversive increase of the average head-to-trunk orientation during contraversive body tilt. On the other hand, the vestibular patients did not show any noticable change of average head-to-trunk orientation. This finding might seem surprising at the first glance but normal head on trunk responses during roll tilt motion in labyrinthine deficient subjects have been observed previously [34]. Overall, the present head measures might be less robust compared to the leg measures because of the additional degrees of freedom of the head during trunk tilt. Slight involuntary yaw or pitch of the head could have biased the detected head tilt in the roll plane. Although the participants were asked to face straight ahead, we refrained from giving too explicit instructions to subjects as we were interested in the spontaneous leg and head responses during passive trunk tilt in the roll plane.

While searching for the mechanisms causing pusher syndrome, previous observations suggested an impaired perception of upright orientation of the body (SPV perception) in stroke patients with pusher syndrome [16]. Using a traditional tilt chair apparatus, Karnath and co-workers showed that pusher patients consciously perceived themselves as upright when they were actually tilted by nearly 20° towards the ipsilateral side. In the present study, we found an ipsiversive tilt of the non-paretic leg in the pusher patients that we recorded in subjects not showing pushing behaviour when they were tilted about 15° to the ipsiversive side. Thus, our present as well as our previous findings [16] argue for an ipsiversive tilt of the SPV between 15° to 20° in pusher patients. Thus, it may be speculated that in pusher patients a representation of own body orientation is disordered that drives both conscious perception of body orientation and automatic postural adjustment of the non-paretic leg during passive body tilt. However, future research is needed to verify this hypothesis by correlating SPV perception with the non-paretic leg orientation in the same sample of pusher patients.

A representation of verticality is necessary for the postural control system to function properly [20,21]. At least three sensory channels are available to estimate the postural orientation of all body segments and the body as a whole: the visual, the vestibular, and the somatosensory system [35]. Depending on body posture and environmental context, afferent somatosensory information might be more dominant than visual and vestibular input to perceive body orientation and to drive the appropriate postural adjustments for stabilizing body posture against external perturbations [24,25,36]. For example, somatosensory afferences have been found to stabilize the head with respect to the trunk during whole body translation and body tilt [24,25,37,38]. Observations in cats before and after bilateral labyrinthectomy also suggested that somatosensory afferences trigger automatic postural responses to translation of the support surface [26-28]. Loss of vestibular input did not affect the spatial and temporal characteristics of the muscle responses or the postural strategy in general, except for a postural hypermetria. Inglis and Macpherson proposed that somatosensory information dominates the body's postural responses when the support surface itself, on which the subject was resting, was stable and contact to the surface well established [27]. The findings of our present study thus let us assume that an ipsiversive deviation of perceived own body orientation results in an ipsiversive bias of (somatosensory triggered) leg posture in patients with pusher syndrome.

Patients with acute unilateral vestibular loss show severe clinical symptoms like vertigo, nausea, postural imbalance and the tendency to fall [39,40]. They also demonstrate disturbed postural control during upright stance and affected postural responses to challenges of body equilibrium following sudden perturbations to the support platform [41]. Also, their perception of the orientation of an external object is compromised as demonstrated by an ipsiversive deviation of the SVV [42]. On the other hand, patients with acute vestibular loss do not show an impaired perception of body orientation [43]. (At most, their perception of body orientation might be slightly impaired in a more dynamic context [44].) The present study has shown that patients with acute unilateral vestibular loss did not exhibit any impairments in their spontaneous leg orientation during passive body tilt; they behaved like non-brain-damaged controls. Thus, it seems that vestibular patients are able to suppress the vestibular imbalance when performing leg adjustments and that disturbed vestibular afferences alone are not responsible for the disordered leg responses seen in pusher patients in the present study. Conversely, stroke patients with pusher syndrome demonstrate almost undisturbed processing of visual and vestibular inputs. Abnormal labyrinthine function does not seem to be a central impairment in pusher patients [45]. In contrast to their markedly disturbed perception of upright body posture, orientation perception of the visual world is unaffected [16,46]. In other words, pusher patients have a severe tilt of body posture, in spite of almost normal visual-vestibular functioning.

The nature of the signal that governs postural adjustment of the legs in sitting humans still is a matter of debate. It was speculated that it might derive from cutaneous pressure sensors. Roll et al. elicited kinesthestic illusions of body lean by vibrating the plantar soles of their subjects [47]. Their interpretation was that cutaneous information from the foot reflects body position as well as state of the body support. In the same sense, skin pressure sensors at the subjects' bottom might be involved in the control of body posture in a seated posture. Alternatively, Forssberg and Hirschfeld applied balance perturbations to sitting subjects and interpreted their findings as evidence for pelvis rotation as the critical stimulus for controlling postural adjustments while being seated [24]. Pelvis rotation might be derived from proprioceptive input of either the lumbar vertebrae or the lower back, hip and abdominal muscles. Finally, truncal graviceptors have been demonstrated to be involved in the perception of body orientation [48]. Interestingly, leg proprioception was not found to influence the perception of body orientation by those truncal graviceptors [48,49]. On the other hand, the control of leg adjustments during trunk tilt in a seated posture must not necessarily be dependent on leg proprioception itself but on a representation of body orientation deriving from different sensory afferences. This hypothesis was supported by Allum et al. who proposed that hip or trunk proprioceptive input drives human balance corrections instead of leg proprioception [50].

Our study revealed that the postural response dynamics of the non-paretic leg were disturbed in pusher patients. The pusher patients' linear regression of the leg-to-trunk showed a steeper slope compared to the subjects not showing pushing behaviour indicating that (with respect to the trunk tilt) their non-paretic leg response was performed stronger than necessary. This finding may imply that in contrast to the subjects without pushing behaviour, pusher patients expressed a stiffened response of their non-paretic leg by keeping their leg almost in a constant orientation with respect to the trunk. The clinical observations as well as the more prominent response of the non-paretic leg might indicate that their postural responses were controlled on an excessive force scale. Models of force control may explain this behaviour by speculating that pusher patients might severely underestimate their own muscle output [51]. This interpretation finds support by observations of Scholle et al. who reported deficits of force control regulation following lesions in both hemispheres [52].

It should be noted that the present groups of subjects could not be perfectly matched regarding all demographic and clinical criteria. Especially, the pusher patients demonstrated a more severe paresis of the contralateral arm and leg. This finding has been reported before [16,18,19,53] and seems to be characteristic for pusher patients. This parameter thus is difficult to counter by any matching procedures. However, we here investigated the patients' *non-paretic* ipsilesional lower extremity and do not expect the severeness of contralateral paresis to determine the characteristics of ipsilateral leg responses to passive trunk tilt. Still, effects of a brain lesion might not be restricted to the contralateral limbs of the body. Noticable changes in motor control parameters of the limbs on the side ipsilateral to the lesion have been reported [54-68]. However, these findings appear to be valid for hemiparetic stroke patients in general and do not seem to be specific to only patients with pusher syndrome, as it was the case in the present study.

## Conclusion

We found disordered spontaneous postural adjustments of the non-paretic leg in response to slow passive body tilt in stroke patients showing pusher syndrome compared to stroke patients without pushing behaviour, patients with acute unilateral vestibular loss, and non brain damaged subjects. The non-paretic leg of the pusher patients showed a constant ipsiversive tilt for an amount which was observed in the non-pusher subjects when they were tilted for about 15° into the ipsiversive direction. Moreover, the balance response of the pusher patients' non-paretic leg was more pronounced compared to subjects without pushing behaviour. Together with the previous finding that pusher patients feel themselves oriented upright when tilted by nearly 20° towards the ipsilateral side [16], the present results may suggest that in pusher patients a representation of own body orientation is disordered that drives both conscious perception of body orientation and automatic postural adjustment of the non-paretic leg during passive body tilt. With respect to clinical practice, the present findings show that the investigation of the patients' leg-to-trunk orientation could serve as an additional bedside tool to detect pusher syndrome in acute stroke patients.

## Competing interests

The author(s) have reported no conflicts of interest.

## Authors' contributions

LJ and HOK participated in all aspects of the study. DB participated in the coordination and data collection of the study. All authors read and approved the final manuscript.

## Pre-publication history

The pre-publication history for this paper can be accessed here:



## Figures and Tables

**Table 1:** Demographic and clinical data of the patients with pusher syndrome, brain damaged patients without pushing behaviour (BD-controls), patients with acute unilateral vestibular loss, and non brain damaged controls (NBD-controls)

			**Pusher**	**BD-Controls**	**Vestibular loss**	**NBD-Controls**
Number			9	9	7	10
Sex			3 f, 6 m	2 f, 7 m	5 f, 2 m	6 f, 4 m
Age	Years	Mean (SD)	69.7 (13.0)	65.1 (15.7)	51.1 (12.3)	63.2 (10.2)
Etiology			7 Infarct 2 Hemorrhage	6 Infarct 3 Hemorrhage	6 Vestib. neuritis 1 Surgery neuroma	
Side of lesion			8 Right/1 Left	5 Right/4 Left	4 Right/3 Left	
Time between onset of disorder and postural examination (d)		Mean (SD)	6.8 (3.1)	5.0 (3.4)	1.8 (1.2)	
SCP-Posture	Sitting	Median (range)	1	0	0	0
	Standing	Median (range)	1	0	0	0
SCP-Extension	Sitting	Median (range)	1 (0.5–1)	0	0	0
	Standing	Median (range)	1 (0.5–1)	0	0	0
SCP-Resistance	Sitting	Median (range)	1	0	0	0
	Standing	Median (range)	1	0	0	0
Visual field defect		% present	22	22	0	0
Paresis of contralesional side		% present	100	89	0	0
Severity	Arm	Median (range)	1 (0–3)	4 (0–5)	5	5
	Leg	Median (range)	1.5 (0–4)	4 (3–5)	5	5
Aphasia		% present	0	44	0	0
Spatial Neglect		% present	88	44	0	0
		% t.n.p.	11	11	0	0

*SCP*: Scale for Contraversive Pushing [15,16]. t.n.p.: testing not possible.

**Table 2:** Effect table for passive trunk tilt

										**Main effects**						
		**Pusher**		**BD-Controls**		**Vestibular loss**		**NBD-Controls**		Group F(3,31)	Viewing condition F(1,31)	Direction F(1,31)	Group × Viewing condition F(3,31)	Group × Direction F(3,31)	Viewing × Direction F(1,31)	Group × Viewing condition × Direction F(3,31)
									
		**Contra**	**Ipsi**	**Contra**	**Ipsi**	**Contra**	**Ipsi**	**Left**	**Right**							

Tilt range [°]	Mean (SD)	32.2 (5.9)	22.7 (10.9)	32.6 (5.6)	35.2 (8.7)	36.6 (10.7)	37.2 (9.7)	43.5 (10.5)	42.0 (9.1)	5.76*	0.06	2.19	0.41	4.08*	0.01	1.362
Tilt frequency [Hz]	Mean (SD)	0.1 (0.04)		0.1 (0.03)		0.1 (0.03)		0.1 (0.03)		1.14	0.24		6.07*			
Max. tilt velocity [°/s]	Mean (SD)	16.5 (7.3)	17.1 (7.8)	21.2 (3.0)	21.2 (2.7)	22.5 (10.7)	22.4 (10.1)	22.8 (8.9)	23.2 (8.6)	1.21	1.35	0.24	0.09	0.47	0.77	1.64

* p < 0.05, two-tailed

**Table 3:** Effect table for the two linear regression parameters of leg orientation as a function of trunk orientation

										**Main effects**						
		**Pusher**		**BD-Controls**		**Vestibular loss**		**NBD-Controls**		Group F(3,27)	Viewing condition F(1,27)	Direction F(1,27)	Group × Viewing condition F(3,27)	Group × Direction F(3,27)	Viewing condition × Direction F(1,27)	Group × Viewing condition × Direction F(3,27)
									
		**Contra**	**Ipsi**	**Contra**	**Ipsi**	**Contra**	**Ipsi**	**Left**	**Right**							

**Intercept of leg-to-trunk function**	Mean (SD)	9.64 (4.72)	10.42 (4.94)	2.09 (5.75)	2.26 (6.02)	0.35 (3.34)	-1.92 (4.02)	2.57 (4.23)	1.61 (5.17)	6.83*	0.19	1.63	0.68	2.00	0.22	2.68
**Slope of leg-to-trunk function**	Mean (SD)	0.86 (0.15)	0.87 (0.26)	0.70 (0.16)	0.63 (0.20)	0.49 (0.19)	0.43 (0.10)	0.55 (0.21)	0.49 (0.22)	9.51**	0.16	1.40	1.04	0.15	1.70	0.18

* p < 0.05, two-tailed; ** p < 0.001, two-tailed

**Table 4:** Effect table for the two linear regression parameters of head orientation as a function of trunk orientation

										**Main effects**						
		**Pusher**		**BD-Controls**		**Vestibular loss**		**NBD-Controls**		Group F(3,28)	Viewing condition F(1,28)	Direction F(1,28)	Group × Viewing condition F(3,28)	Group × Direction F(3,28)	Viewing condition × Direction F(1,28)	Group × Viewing condition × Direction F(3,28)
									
		**Contra**	**Ipsi**	**Contra**	**Ipsi**	**Contra**	**Ipsi**	**Left**	**Right**							

**Intercept of head-to-trunk function**	Mean (SD)	-0.54 (5.23)	0.41 (4.87)	-2.29 (5.10)	-2.65 (4.90)	-0.98 (4.67)	-0.41 (5.09)	5.05 (3.12)	4.80 (3.93)	4.68*	0.52	0.80	1.23	1.50	1.42	2.01
**Slope of head-to-trunk function**	Mean (SD)	1.04 (0.27)	1.20 (0.23)	0.94 (0.18)	0.90 (0.22)	0.85 (0.16)	0.78 (0.16)	0.90 (0.36)	0.92 (0.31)	1.80	7.70*	0.55	1.34	2.89*	0.39	1.36

* p < 0.05, two-tailed
